# Controls on microbial response above typical oil and gas reservoirs in the Junggar Basin and implications for exploration

**DOI:** 10.3389/fmicb.2026.1979085

**Published:** 2026-10-21

**Authors:** Li Ding, Linfeng Wu, Shuai Luo, Yubing Wu, Hui Wu, Hai Mei

**Affiliations:** 1 Peking University Ordos Research Institute of Energy, Ordos, China; 2 Inner Mongolia Industrial Technology Engineering Center of Carbon Capture, Utilization and Storage, Ordos, China; 3 School of Earth and Space Sciences, Peking University, Beijing, China; 4 AE&E Institute, Beijing, China

**Keywords:** butane-oxidizing bacteria, controlling factors, hydrocarbon prediction, light hydrocarbon microseepage, microbial hydrocarbon exploration

## Abstract

**Introduction:**

Microbial hydrocarbon exploration, which is based on the theory of light hydrocarbon microseepage, has played an increasingly important role in oil and gas exploration. However, the microbial response characteristics above different types of hydrocarbon reservoirs and their geological controlling factors remain poorly understood, severely constraining the predictive effectiveness of microbial prospecting.

**Methods:**

In this study, butane-oxidizing bacteria were selected as indicator microorganisms to systematically investigate their distribution characteristics above structural, lithologic, and stratigraphic hydrocarbon reservoirs in the Junggar Basin and to elucidate the dominant geological controls. A total of 1,607 soil samples were collected at depths of 20–25 cm from two study areas. Microbial values (MV) were determined using a gradient dilution–selective cultivation–colony counting method. Combined with correlation analysis and seismic profile overlay, the relationships between MV anomalies and geological conditions of hydrocarbon reservoirs were established.

**Results and discussion:**

The results indicate that the microbial anomaly distributions are consistent with the predominantly near-vertical migration theory of light hydrocarbons. Structural reservoirs exhibit large-scale, high-intensity MV anomalies, which closely coincide with anticline cores. Lithologic reservoirs are characterized by irregular, band-shaped MV anomalies, whereas stratigraphic reservoirs show clustered anomalies near pinch-out zones. The mean MV is strongly positively correlated with hydrocarbon enrichment (*R*^2^ = 0.8035) but only weakly correlated with reservoir pressure (*R*^2^ = 0.0308) and crude oil density (*R*^2^ = 0.0515). Light hydrocarbons in the reservoir could still migrate to the surface even when covered by thick mudstone caprocks. Hydrocarbon enrichment in subsurface reservoirs plays a critical role in shaping the surface distribution of hydrocarbon-oxidizing bacteria. The microbial response models established for different types of hydrocarbon reservoirs provide robust theoretical support and technical references for hydrocarbon prediction in geologically complex areas.

## 1 Introduction

Microbial hydrocarbon exploration is based on the theory of light hydrocarbon microseepage. By collecting surface soil or shallow marine sediment samples and applying geomicrobiological techniques, specific hydrocarbon-indicating microorganisms can be detected to predict the presence and spatial distribution of underlying hydrocarbon reservoirs (Wu et al., 2014; Zhou et al., 2020). The enrichment of hydrocarbon-indicating microorganisms in soils is closely related to the metabolic utilization of light hydrocarbons migrating to the surface, while the characteristics of light hydrocarbon microseepage are fundamentally controlled by geological conditions (Klusman, 2015).

With respect to the surface response patterns of light hydrocarbon microseepage, traditional surface geochemical methods generally suggest that the upward migration of light hydrocarbons produces halo-shaped or apical anomalies above hydrocarbon reservoirs (Tang et al., 1998; Liu et al., 1999). In contrast, Kartsev et al. (1959) argued that microbial prospecting typically produces apical anomalies and that the halo anomalies occasionally observed are often associated with major fault systems. As noted by Siegel (1974), “it is noteworthy that some other potential methods for hydrocarbon detection also exhibit halo-type anomalies (e.g., radioactivity, conductivity, and resistivity), whereas microbial methods are exceptional in that the entire target area tends to appear anomalous.” When methane-oxidizing bacteria and hydrocarbon-oxidizing bacteria are used as indicators, Yang et al. (2017) demonstrated a strong correspondence between microbial anomalies and producing wells in the Chenjiazhuang Oilfield of the Bohai Bay Basin, confirming that microbial responses above light hydrocarbon microseepage zones are predominantly characterized by apical anomalies.

Although hydrocarbon-indicating microorganisms generally display apical anomaly patterns above hydrocarbon accumulation, variations in geological conditions may lead to distinct microbial response modes. One of the most debated aspects of the microseepage theory concerns the verticality of hydrocarbon migration. Some researchers argue that faults serve as primary migration pathways for hydrocarbons; therefore, microseepage should preferentially occur along faults or unconformities through lateral migration rather than near-vertical ascent. Other studies suggest that pervasive microfracture systems within overlying strata constitute the dominant conduits for the near-vertical migration of light hydrocarbons (Saunders et al., 1999; Klusman, 2011). Wang et al. (2018), on the basis of the investigations above on fault-block reservoirs, proposed that although faults represent preferential pathways for hydrocarbon migration, microfractures also play important roles. The effectiveness of a fault as a microseepage conduit is determined primarily by its sealing properties rather than its geometric attributes.

Rosaire (1940) and Jones and Drozd (1983) recognized the distinction between macroseepage and microseepage, whereas Abrams (2005) provided quantitative definitions of their characteristic differences. Macroseepage refers to visible hydrocarbon migration along dominant pathways, such as faults and unconformities, and is typically expressed as linear or band-shaped anomalies. In contrast, microseepage involves the near-vertical upward migration of light hydrocarbons through microfractures, resulting in the formation of blocky or clustered anomaly patterns that are not visually detectable. Through case studies in the Sanhu area of the Qaidam Basin and the Zhenba area of the Sichuan Basin, Zhang et al. (2009, 2010) further confirmed these distinctions.

In addition to migration pathways, other geological factors may significantly influence microseepage intensity. Yuan et al. (2009) demonstrated that a decrease in reservoir pressure leads to a reduction in seepage flux, particularly near wellbores where the decline is most pronounced. Richers et al. (1982) reported that continuous water and gas injection during field development in the Patrick Draw Oilfield (USA) gradually restored reservoir pressure, resulting in a substantial increase in oil production as well as a marked increase in soil free gas concentrations above the reservoir. Tang et al. (2019) suggested that caprock porosity also affects microseepage fluxes. However, owing to the complexity of interactions between hydrocarbon seepage and geological conditions, quantitative evaluations remain challenging, and systematic investigations of these controlling factors are still limited.

In summary, previous studies generally indicate that hydrocarbon-indicating microorganisms above oil and gas fields predominantly exhibit apical anomalies, whereas macroseepage along faults may generate localized point or linear anomalies. Given the complexity of geological settings and the diversity of trap types, the planar distribution patterns of microbial data can vary significantly across regions. However, systematic syntheses of microbial distribution patterns above typical structural, lithologic, and stratigraphic reservoirs are still lacking, and corresponding geological models that integrate theory with field observations remain underdeveloped. The establishment of surface microbial response models for different reservoir types is therefore expected to provide valuable guidance for hydrocarbon prediction.

Moreover, long-term studies on microseepage mechanisms have focused primarily on the migration phase, rate, and verticality of light hydrocarbons, whereas the controlling effects of geological factors such as hydrocarbon abundance, reservoir pressure, and fluid properties have received less attention. Geological heterogeneity among different reservoir types (structural, lithologic, and stratigraphic) may result in distinct microbial response mechanisms. Existing studies are often limited to single reservoir types and lack cross-type comparisons and quantitative discrimination of dominant controlling factors.

In this study, butane-oxidizing bacteria were selected as indicator microorganisms. Taking representative structural, lithologic, and stratigraphic hydrocarbon reservoirs in the Junggar Basin as case studies, we investigate the distribution characteristics of butane-oxidizing bacteria above these reservoirs. The relationships between microbial data, anomaly patterns, and geological conditions are analyzed to evaluate the individual effects of hydrocarbon abundance, reservoir pressure, hydrocarbon properties, migration pathways, and caprock characteristics. The microbial response patterns and geological influencing factors above typical reservoirs are identified, providing both a theoretical basis and practical insights for microbial hydrocarbon prediction.

## 2 Materials and methods

### 2.1 Geological setting

This study was conducted in the Junggar Basin, northwestern China. Structural reservoirs were investigated in the Gaoquandong Anticline within the Sikeshu Depression along the southern margin of the basin, while lithologic and stratigraphic reservoirs were examined on the eastern slope of the Fukang Depression (Figure 1). These two areas were selected to construct microbial response models above different reservoir types.

**Figure 1:**
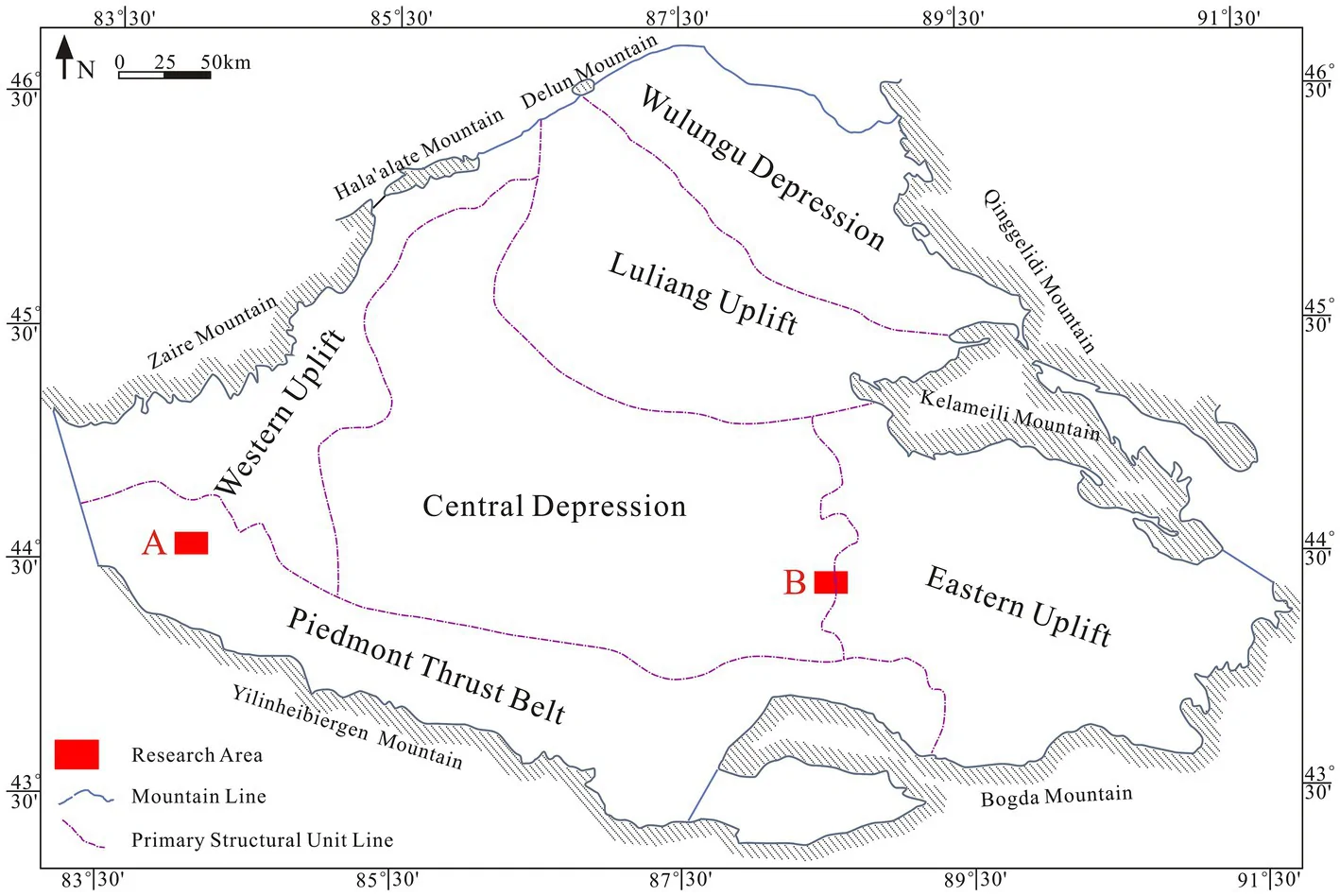
Tectonic subdivision of the Junggar Basin and location of the study area. The base map was provided by the Exploration and Development Research Institute of PetroChina Xinjiang Oilfield Company, with the study area marked by the authors.

#### 2.1.1 Study area A: Sikeshu depression, southern Junggar Basin

The Sikeshu Depression is located at the intersection of the foreland thrust belt of the northern Tianshan Mountains and the northwestern structural system of the Junggar Basin, forming a key secondary tectonic unit at the western end of the southern basin margin (Gao et al., 2013). The depression experienced three major tectonic stages—Indosinian, Yanshanian, and Himalayan—resulting in a dual structural framework characterized by “faulting at depth and flexural subsidence upwards.” The deeper structural system consists of Mesozoic strike-slip faults and en echelon folds, whereas the shallow part is dominated by Cenozoic detachment thrust structures. Strong northward thrusting of the northern Tianshan Mountains during the Himalayan period played a dominant role in shaping the present structural configuration (Zhao et al., 2022).

The depression contains a complete sedimentary succession from the Permian to the Quaternary. The Jurassic Badaowan Formation and the Palaeogene Anjihaihe Formation constitute two main source rock intervals. The former consists mainly of lacustrine mudstones and coal-bearing strata, whereas the latter is composed of saline lacustrine dark mudstones. High-quality reservoirs are present in the Cretaceous Qingshuihe Formation and the Neogene Shawan Formation, whereas thick mudstones of the Palaeogene Anjihaihe Formation serve as regional caprock (Gao et al., 2013; Du et al., 2019).

Sedimentary evolution in the depression is closely coupled with tectonic activity. During the Jurassic, lacustrine–deltaic environments prevailed. In the Cretaceous transgressive stage, high-quality stacked sand bodies of the Qingshuihe Formation developed under the control of palaeogeomorphic slope breaks. During the Palaeogene–Neogene period, braided delta–saline lake depositional systems were dominant, with sediment supply shifting from the southern Tianshan Mountains in the early stage to increasing contributions from the Zaire Mountains in the northwest during the later stage (Yu et al., 2023). The depression is characterized by multiple hydrocarbon sources, multistage charging, and vertically stacked petroleum systems. Unconformities and faults serve as key pathways for near-vertical hydrocarbon migration (Du et al., 2019).

The Gaoquan structural belt experienced three major hydrocarbon charging episodes: low-maturity oil at ~16 Ma, mature oil at ~7 Ma, and natural gas at ~3 Ma. Late-stage gas charging plays a decisive role in hydrocarbon accumulation (Gui et al., 2023). In 2018, well GT1 was drilled, targeting the lower petroleum system beneath the Gaoquandong Anticline, with a designed depth of 5,980 m (Du et al., 2019).

#### 2.1.2 Study area B: eastern slope of the Fukang Depression

The eastern slope of the Fukang Depression is located in the eastern part of the Central Depression of the Junggar Basin. It is bounded by the North Santai Uplift to the north and the southern Junggar foreland thrust belt to the south, forming the core unit of a “two depressions flanking one uplift” structural framework. Nose-shaped structural belts and associated fault systems constitute the fundamental framework for hydrocarbon migration and accumulation (Ding et al., 2017; Guo et al., 2024).

The Jurassic Shishugou Group (including the Toutunhe and Qigu formations) represents the principal exploration interval. Depositional systems are dominated by deltaic–lacustrine environments, with subaqueous distributary channels in delta fronts and nearshore–shallow lacustrine sand bars as the main reservoir microfacies. The sediments were sourced mainly from the northern Karamay Mountains and the North Santai Uplift, with depocenters aligned along the axes of nose-shaped structures (Wei et al., 2023).

The reservoir rocks are predominantly low-maturity fine- to fine–medium-grained feldspathic lithic sandstones. Calcite and anhydrite cements are well developed, and the distribution of anhydrite is controlled by deep faults and closely associated with hydrocarbon shows (Luo et al., 2026). The Shishugou Group exhibits a “lower-generation, upper-reservoir, fault–sand coupling” accumulation pattern. Hydrocarbons generated from high-quality source rocks of the Permian Lucaogou Formation migrated and accumulated through fault–sand transport systems. Favorable accumulation zones are controlled by nose-shaped structures, and effective spatial coupling between faults and sand bodies is critical for efficient hydrocarbon accumulation, resulting in the formation of lithologic–structural composite reservoirs (Ding et al., 2017; Guo et al., 2024). Geological investigations indicate that fluvial sand bodies of the Jurassic Toutunhe Formation—a lithological trap—develop in the western part of Study Area B. Affected by structural uplift, the Permian–Jurassic strata in this area are successively eroded and pinch-out from west to east, forming a series of stratigraphic unconformity traps beneath the Cretaceous unconformity. In the eastern part of Study Area B, stratigraphic unconformity traps have developed near the truncation line of the Qigu Formation within the Jurassic Shishugou Group.

### 2.2 Field survey design and sampling

The grid sampling design was applied in this study to improve the detection of light hydrocarbon microseepage. The sampling grid spacing was determined according to regional geological conditions and trap types. Sampling in Study Area A followed a uniform grid pattern with a spacing of 500 m × 500 m. A total of 256 microbial samples were collected. Study Area B was also sampled using a uniform grid design, covering an area of 131.5 km^2^ with a grid spacing of 330 m × 330 m. A total of 1,351 microbial samples were collected. As in previous research (Ding et al., 2026), sampling points should be selected to avoid areas subject to human interference—such as roads, oil wells, and residential areas—as well as special environments like lakes. If suitable samples cannot be collected within a 5-m radius of the designated point, the sampling point should be shifted outward, with the shift distance being less than half the grid spacing. For each sampling point, three soil subsamples were collected at 5-meter intervals, then mixed to form a composite sample. Soil samples were collected from depths of 20–25 cm, and each sample weighed no less than 150 g. Sample pretreatment was performed on the same day after collection to stabilize the microorganisms in the samples and avoid interference caused by environmental changes.

### 2.3 Laboratory analysis

Butane-oxidizing bacteria can serve as specific microbial indicators for oil and gas exploration, as the butane source is specific to oil and gas, and light hydrocarbon microseepage enables abnormal enrichment of butane-oxidizing bacteria in surface soi (Deng et al., 2016, 2018). The detection of butane-oxidizing bacteria was performed by plate culture using selective medium. While the toxicity of butanol inhibits the growth of general microorganisms, it serves as an intermediate metabolite of butane-oxidizing bacteria and provides the carbon source necessary for their growth. Therefore, butanol-containing medium enables selective cultivation (Zhang et al., 2013). The selective medium used was based on an inorganic salt medium containing agar, sterilized by autoclaving at 121 °C for 20 min, with 1-butanol added as the single carbon source. Microbial culture and detection were conducted following four main steps (Ding et al., 2026). Preliminary tests: A subset of soil samples was subjected to gradient dilution experiments to determine the optimal dilution factor (Study Area A: 4,100-fold; Study Area B: 100-fold in farmland, 10-fold in Gobi). Plate inoculation: 5 g of each soil sample was thoroughly mixed with nutrient solution. On the basis of the selected dilution factor, inoculate 0.1 mL of the diluted solution onto the selective medium. Incubation: Inoculated plates were incubated at a constant temperature of 38 °C for 7 days in a biochemical incubator (Boxun SPX-250BZ, China). Colony counting: After incubation, single colonies appear as circular, translucent to opaque spots 1–2 mm in diameter, primarily milky white, pale yellow, or light off-white in color, with surfaces smooth and moist. The colonies were counted using an electronic colony counter (Shineso Icount30D, China). Three parallel plates were prepared for each sample. Additionally, positive controls, negative controls, and blank controls were included to ensure the validity of the medium.

Microbial values (MV) obtained by counting the number of colonies on the plates. Since the counter tallies the number of hydrocarbon-oxidizing bacteria in each plate, the unit of measurement is Colony-Forming Units (CFU) per plate, hereinafter referred to as CFU. The MV reflects the intensity of hydrocarbon seepage from active underlying petroleum systems and serves as a quantitative indicator for identifying the presence and spatial extent of hydrocarbon reservoirs (Zhang et al., 2024). The smoothed microbial value (SMV) was defined as the average MV of three adjacent sampling points along a profile. The MV above the well was defined as the average MV from nine sampling points near the well.

The landform of Study Area A consists predominantly of farmland, while Study Area B is characterized by dominant Gobi in the north and alternate distribution of farmland and Gobi in the south. The soil types are dominated by sandy loam and silty loam, with minor occurrences of sand and clay. When distinguishing hydrocarbon microleakage signals from shallow environmental background effects, the selection of sampling depth and the identification of vadose zone interference are critical (Zhou et al., 2020; Atwah et al., 2024). Therefore, based on the environmental impact factor correction method established in our previous research (Ding et al., 2026), landforms and salinity were identified as the primary factors. Consequently, the sampling depth and microbial detection dilution ratio were optimized, and the obtained microbial data were normalized between zones. Specifically, different detection dilutions were applied to the farmland and Gobi samples from Study Area B, and threshold values were then determined separately. Taking the threshold value from the Gobi area with larger sample sizes as the baseline, the ratio of threshold values between farmland and Gobi areas was defined as the correction coefficient. The microbial data from farmland were then mapped to the same scale as the Gobi area using this correction coefficient. It should be clarified that the normalization process does not alter the trends in microbial anomaly distribution across two areas. After environmental correction, the microbial anomaly distribution aligns with the hydrocarbon potential of known wells. However, this method cannot completely exclude shallow environmental heterogeneity, as it assumes that the relationship between MV and hydrocarbon microseepage under the two landform types can be approximately aligned using the threshold ratio. Future research should combine multi-depth sampling, multi-parameter monitoring, and isotope tracer verification to more reliably extract microseepage signals related to deep hydrocarbon reservoirs (Atwah et al., 2024; Xu et al., 2024).

### 2.4 Data analysis

The MV was classified into five levels for interpretation and visualization. Ultrahigh, high, and moderate anomalies are represented by red, orange, and yellow, respectively, whereas low anomalies and background values are indicated by green (or white) and blue, respectively.

The threshold separating anomalous and background MV can be determined using methods such as statistical analysis, frequency histogram or forward modelling. In the absence of suitable calibration wells for forward modelling, the statistical and frequency histogram approach were adopted to preliminarily define microbial thresholds (Ding, 2024). The lower limit of microbial anomalies using the statistical approach was calculated using the following equation:

$$V_{0}=X+\mathrm{KS}$$

where *V*_0_ is the lower threshold of microbial anomalies (i.e., the boundary between yellow anomalous values and green low-anomaly values, CFU); *X* is the mean background MV (i.e., the mean value of microbial measurements below the overall mean, CFU); *K* is a coefficient typically ranging from 1.0 to 3.0 to ensure a high confidence level, with values empirically set within this range depending on the distribution characteristics of microbial data in study area; and *S* is the standard deviation (i.e., the standard deviation of microbial measurements below the overall mean).

The frequency histogram method involves plotting frequency histograms of microbial data to observe the distribution characteristics and determine the threshold between anomalous and background values (Abrams, 2005). Ideally, the frequency distributions of both the background and anomalous bands in microbial data exhibit characteristics of a normal distribution, presenting a “bimodal” shape. However, in actual exploration, due to the heterogeneity of geological conditions, the distributions of background values and anomalous values may overlap, with their intersection point—also known as a “breakpoint”—typically serving as the threshold between anomalous and background values.

The spatial distribution map of MV in study areas was generated using Surfer software, based on Kriging spatial interpolation of microbial data. A regular rectangular grid of 288 columns × 166 rows was constructed, with grid step sizes of 82.42 m and 82.38 m in the X and Y directions, respectively (approximately one-quarter of the sampling interval). A linear variate model was selected for interpolation, without introducing a trend term. A circular search strategy was adopted with a search radius set at 13,600 m, and the search area was divided into 4 sectors for neighborhood sampling. Interpolation neighborhood constraints: For a single-point estimate, a maximum of 64 neighboring samples may be selected globally, with a maximum of 16 samples per sector; at least 8 samples are required to perform valid interpolation. When the number of empty sectors exceeds 3, the corresponding grid nodes are automatically set to zero to ensure the reliability of the interpolation results.

In evaluating the impact of geological factors on MV, the study used individual wells as the unit of analysis; the mean MV for each well was calculated based on the nine sampling points above it. Geological information included cumulative test production, formation pressure coefficient, and crude oil density. As the survey work in Study Area A and B differed in both sampling period and detection method, resulting in significant differences in the absolute values of microbial counts, a direct comparison is not appropriate. Therefore, the microbial detection results for both areas should be discussed separately. Ordinary Least Square regression was used for data fitting analysis. The robustness of regression model was assessed using the residual bootstrap method (*B* = 2000) and leave-one-out cross-validation. Additionally, spatial autocorrelation testing of the regression residuals was performed by constructing a KNN spatial weight matrix based on well coordinates. All of the above analyses were conducted in R4.6.1.

## 3 Results

### 3.1 Surface microbial response characteristics above structural trap

Statistical analysis revealed that the mean background concentration of hydrocarbon-oxidizing bacteria in Study Area A is 92 CFU, with a background standard deviation of 39 CFU. When the coefficient *K* is set to 1.5, the statistically determined lower threshold for microbial anomalies is 150 CFU. Based on the frequency distribution characteristics of MV in this area, the threshold separating anomalous from background values was calculated as 149 CFU. Since there were no suitable known wells nearby to serve as calibration wells, the microbial anomaly threshold was ultimately determined to be 149 CFU by combining the two methods described above. The detection results reveal that high and ultrahigh microbial anomalies are predominantly distributed over the structural high of the northern Gaoquandong structure, corresponding to the location of Well GT1 (Figure 2). Within the anomalous zone of the Gaoquandong Anticline in the Sikeshu Depression, 32 samples were analyzed, covering an area of approximately 8 km^2^. The mean MV within the anomalous zone is 209 CFU, which is significantly greater than the threshold value of 149 CFU, and anomalous samples account for 91% of the total samples in that zone. These results indicate a strong intensity of light hydrocarbon microseepage at the structural high of the northern trap.

**Figure 2:**
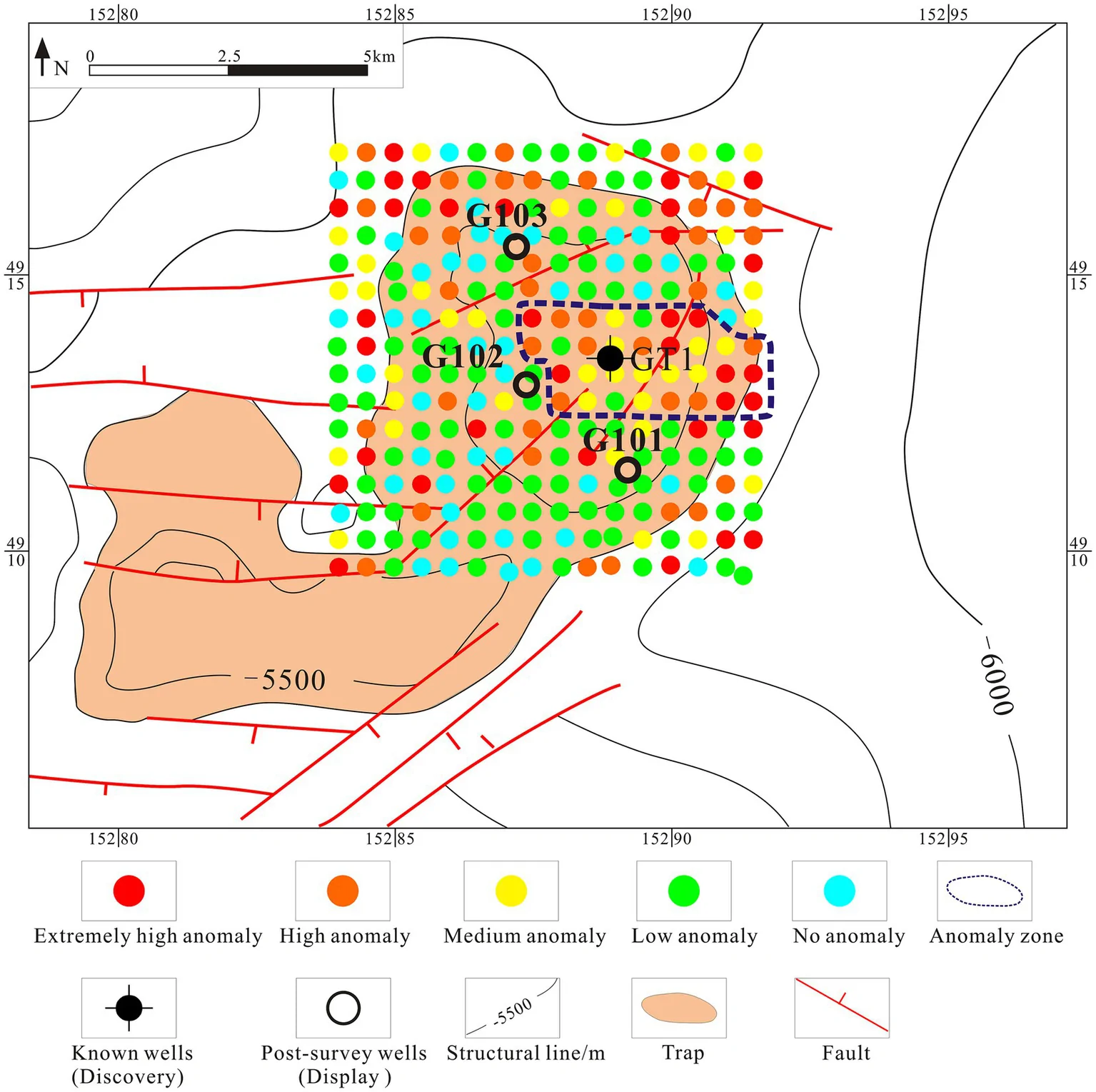
Plane distribution map of MV in the Sikeshu Depression of the Junggar Basin. The GT1 well drilled within the microbial anomaly zone encountered industrial oil and gas flow discovery, whereas the three wells located in low-value and background zones were unsuccessful.

In contrast, within the northern trap but outside the microbial anomaly zone (within the 5,500 m structural contour), the mean MV decreases to 124 CFU, and only 28% of the samples exceed the anomaly threshold. This suggests that the northern trap is not uniformly hydrocarbon-bearing and that the structurally low positions are dominated by low or background MV, implying relatively high exploration risk.

Drilling of Well GT1 yielded high-rate industrial oil and gas flow. Post-drilling analysis indicates that the reservoir encountered by Well GT1 is a layered anticline reservoir, with a reservoir pressure of 88.25 MPa and an interpreted porosity of 18% in the Qingshuihe Formation. In the southern Junggar Basin, the Cretaceous Tugulu Group contains 500–2,000 m thick mudstones that act as regional caprocks (Du et al., 2019). The pressure coefficient of the Tugulu Group in Well GT1 reaches 2.2, indicating excellent sealing capacity (Zhuo et al., 2020).

### 3.2 Surface microbial response characteristics above lithologic trap

The threshold value for the eastern slope of the Fukang Depression (Study Area B) was determined using the previously described method, in which statistical analysis yielded a threshold of 30 CFU (*X* = 15 CFU, *S* = 12 CFU, *K* = 1.25), the frequency distribution indicated a threshold of 29 CFU, and the forward modeling wells showed a threshold ranging from 22 (FD051) to 32 (FD2) CFU. Integrating these results, the microbial anomaly threshold was finalized at 30 CFU. The planar microbial prediction results (Figure 3) delineate three irregular, band-shaped favorable zones within the study area, corresponding to the FD2, FD5, and FD8 well zones. These favorable belts exhibit an overall northwest–southeast orientation, which is consistent with the structural trend of the Toutunhe Formation. This spatial correspondence suggests that lithologic traps within the Toutunhe Formation may have favorable hydrocarbon accumulation potential. High microbial anomalies were detected above the drilled discovery wells FD2, FD5, and FD8, whereas low or background MV were observed above the two display wells FD021 and FD051. Overall, the microbial detection results are in good agreement with the drilling outcomes from the five known wells.

**Figure 3:**
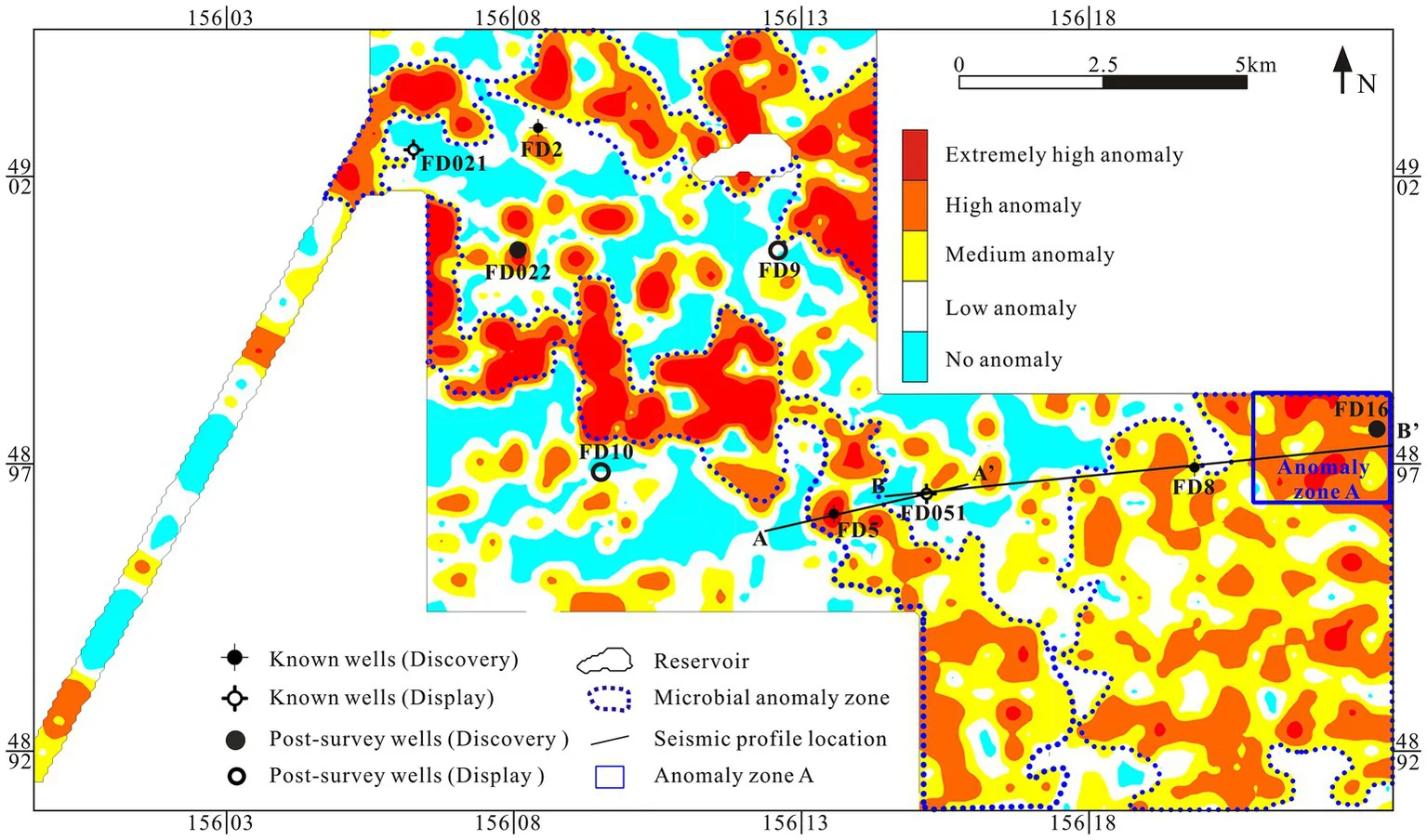
Achievements of microbial hydrocarbon prediction using the Kriging interpolation method in the eastern slope of the Fukang Depression.

The interwell seismic profiles from the legacy 3D seismic data (Figure 4A) and newly acquired high-resolution 3D seismic data (Figure 4B) are compared in Figure 4 with the MV (the profile A–A’ locations are shown in Figure 3). Although Wells FD051 and FD5 are less than 2 km apart, they exhibit markedly different microbial responses. The mean MV above Well FD051 is 22 CFU, which is below the threshold, whereas the mean value above Well FD5 is 48 CFU, which exceeds the threshold. On the basis of interpretations of the legacy 3D seismic data acquired in 2010, Wells FD051 and FD5 were initially considered to penetrate the same sand body, with FD051 located at a structural high and FD5 at a structural low. However, the drilling results revealed water production at the structural high and oil production at the structural low, resulting in an apparent inversion of the oil–water distribution that contradicts classical petroleum geological principles. In 2016, newly acquired high-resolution 3D seismic data revealed that Wells FD051 and FD5 actually target different lithologic traps. Well FD5 penetrates a lower-positioned lenticular lithologic reservoir within the Toutunhe Formation, whereas Well FD051 targets the lower part of a different sand body updip. As the two wells intersect distinct lithologic traps, the observed oil–water inversion can be reasonably explained. High MV detected above Well FD5, combined with its successful hydrocarbon production, further indicate that light hydrocarbon microseepage above lenticular lithologic reservoirs is consistent with predominantly near-vertical migration, and that the enrichment of hydrocarbon-oxidizing bacteria is related to hydrocarbon accumulation in reservoir.

**Figure 4:**
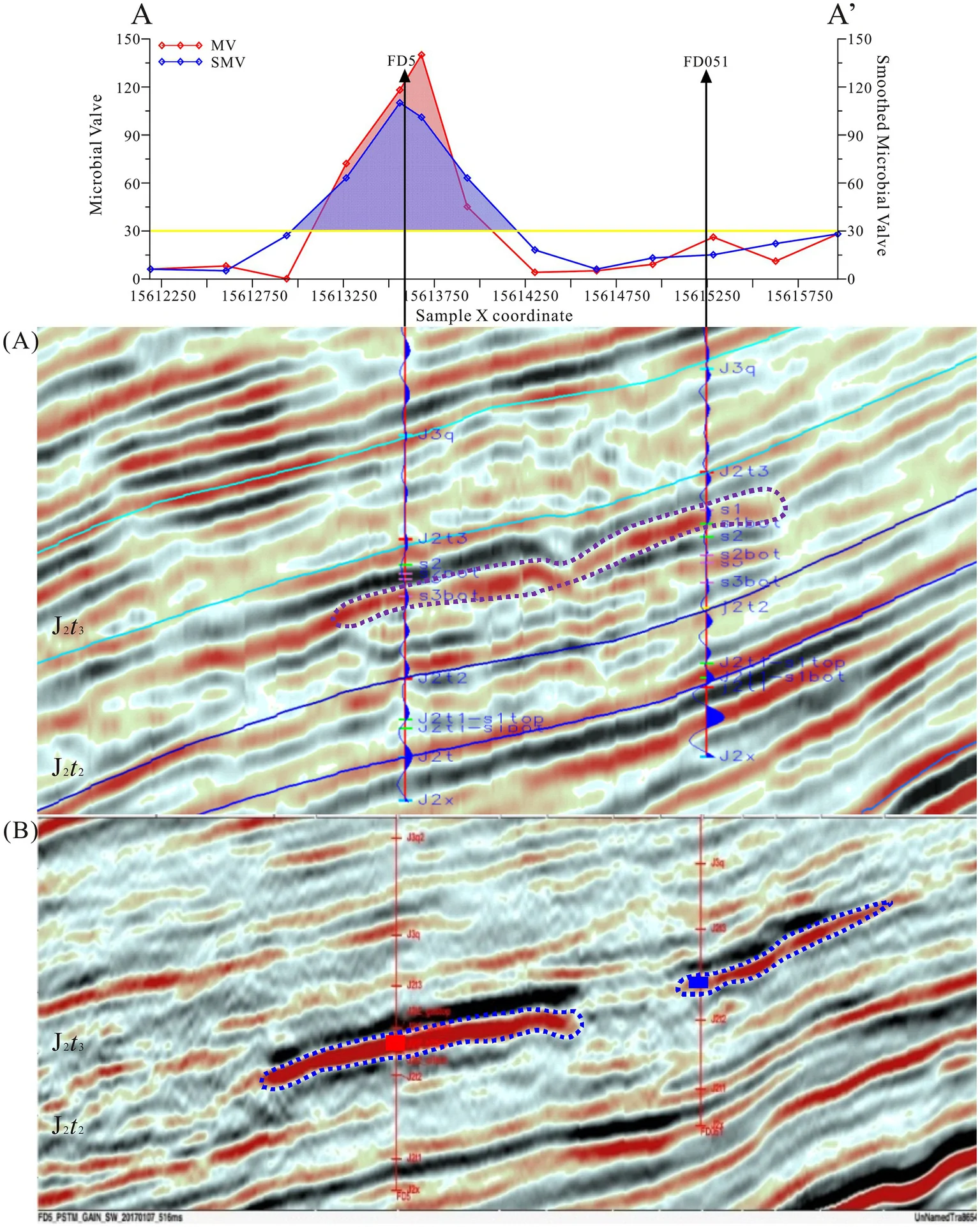
Comparison between legacy 3D **(A)** and newly acquired 3D **(B)** interwell seismic profiles, along with their correspondence to MV and SMV (Average MV at three adjacent sampling points along the profile).

### 3.3 Surface microbial response characteristics above stratigraphic trap

Microbial prospecting results on the eastern slope of the Fukang Depression indicate that Anomaly Zone A, located northeast of the known discovery Well FD8, represents one of the most promising exploration targets in the area. The anomalous zone covers approximately 8 km^2^ and contains 52 anomalous MV points, including 30 high and extremely high anomalies. These anomalies exhibit clustered and continuous spatial patterns, extending eastwards from Well FD8 along the structural slope towards higher structural positions, reflecting strong light hydrocarbon microseepage.

The east–west seismic profile B–B′, overlaid with MV (Figure 5), shows that no significant seismic anomalies are present in the Jurassic Toutunhe Formation beneath Anomaly Zone A. However, a prominent seismic anomaly is identified in the Qigu Formation beneath the Cretaceous Tugulu Group (K₁tg) unconformity. This anomaly is characterized by continuous high-amplitude peak reflections, which pinch out updip at the K₁tg unconformity and gradually weaken downdip towards Well FD8 while maintaining lateral continuity. This seismic feature represents a stratigraphic unconformity controlled by the pinch-out boundary of the Qigu Formation, with a closure height of approximately 460 m and a trap area of approximately 6 km^2^. The strongest microbial anomalies occur above the pinch-out zone and gradually decrease towards lower structural positions.

**Figure 5:**
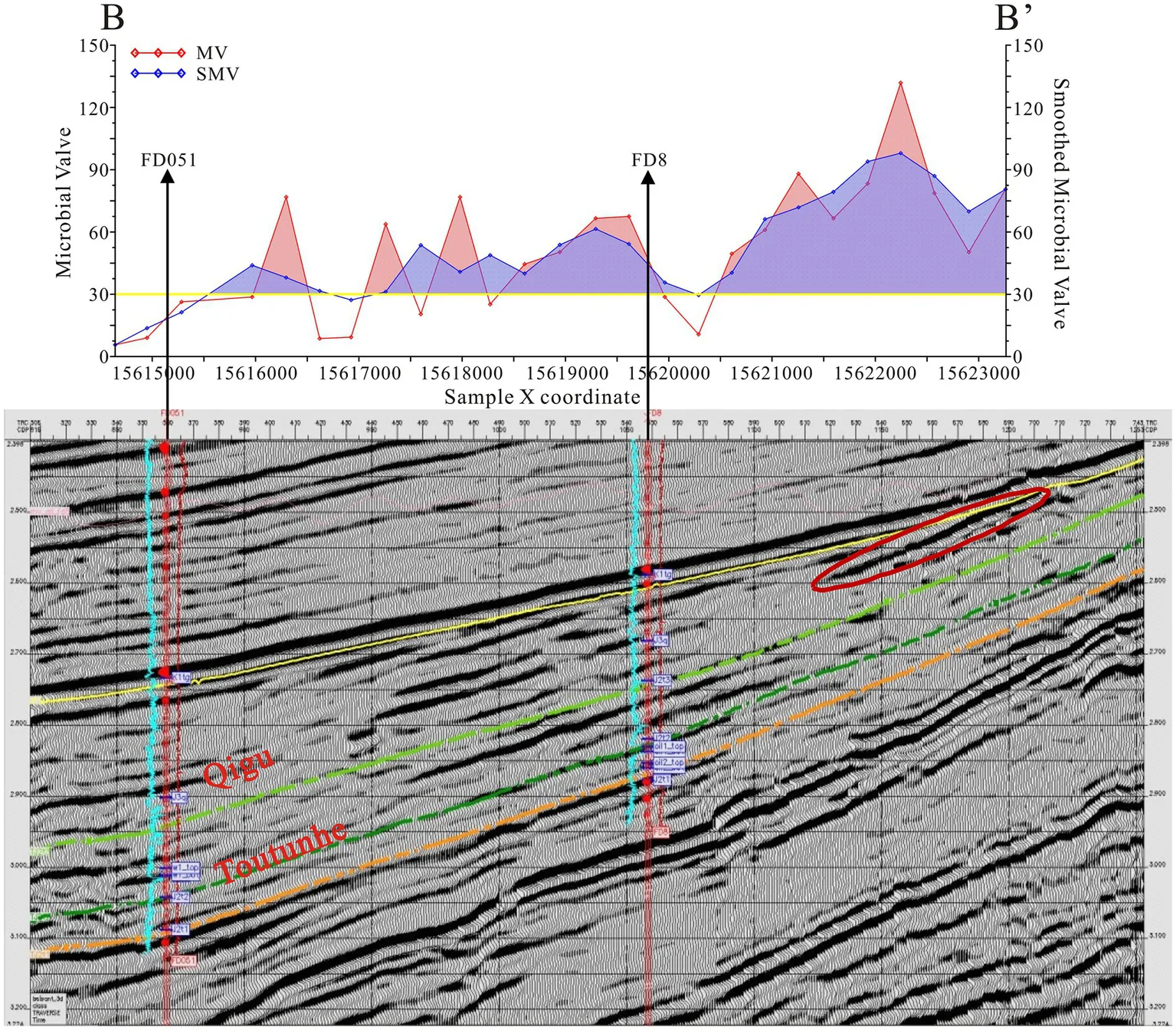
Overlay of microbial anomalies (including the distribution of MV and SMV) and the FD051–FD8 seismic profile.

## 4 Discussion

### 4.1 Comparison of microbial response models for typical trap types

On the basis of the analysis of geological characteristics and microbial hydrocarbon detection results in the Sikeshu Depression of the Junggar Basin, a microbial response model for typical anticlinal structural hydrocarbon reservoirs is summarized (Figure 6A). This model indicates a strong near-vertical correspondence between the distribution of hydrocarbon-oxidizing bacteria and structural geometry. Microbial anomalies are predominantly developed over the main structural highs, characterized by laterally continuous, high-intensity, cluster-like distributions with a high proportion of anomalous values. These anomalies show good spatial agreement with structural culminations, and the drilling results confirm significant hydrocarbon enrichment in the underlying reservoirs. In contrast, the MV over the structural flanks and low positions are generally low or at background levels, which is consistent with the poor hydrocarbon potential confirmed by drilling. The microbial response pattern above anticlinal reservoirs conforms well to the fundamental characteristics of light hydrocarbon microseepage.

**Figure 6:**
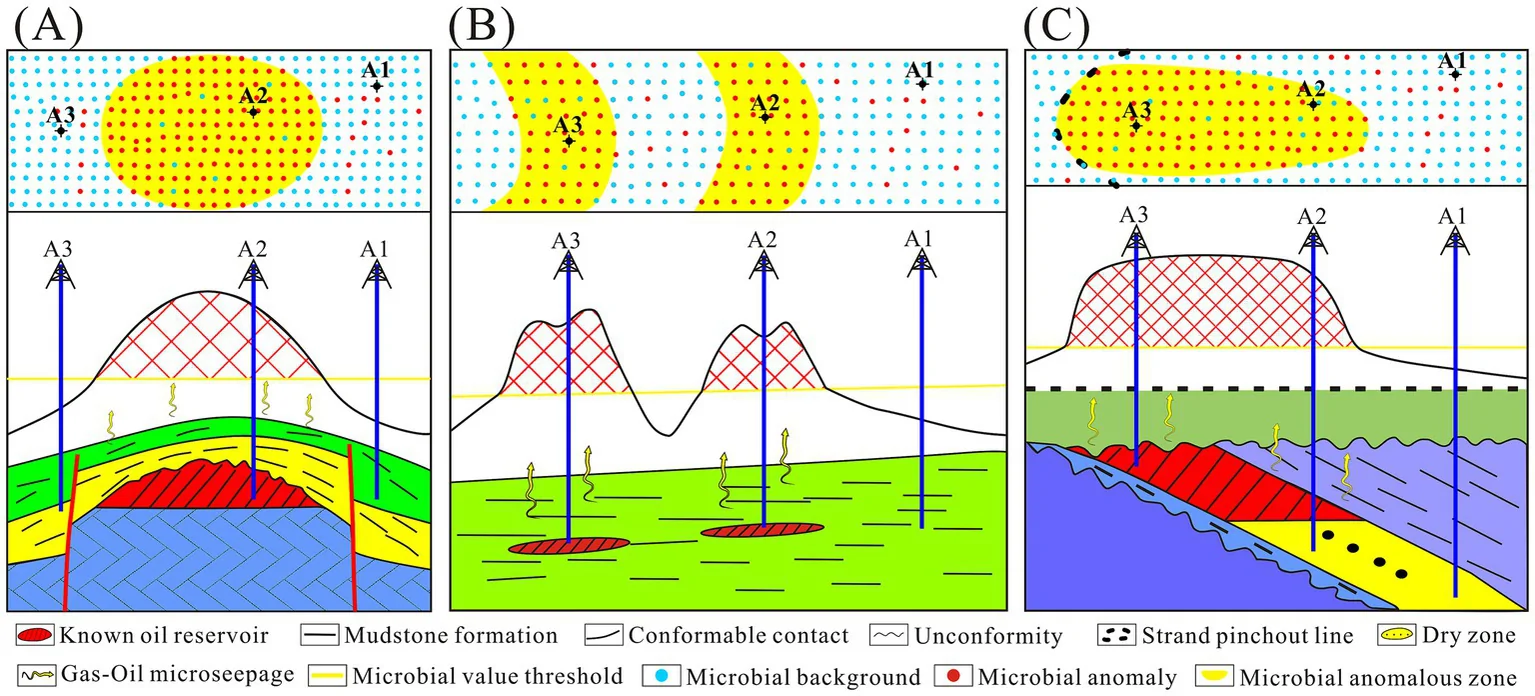
Geological models of microbial responses to typical hydrocarbon traps: **(A)** typical anticline-type structural reservoir; **(B)** lithologic reservoir; **(C)** typical unconformity-related reservoir.

This study further demonstrates that hydrocarbon reservoirs buried at depths greater than 5,000 m can still be detected at the surface using microbial prospecting methods; even in the presence of thick regional mudstone cap rocks (500–2,000 m), light hydrocarbon microseepage can be detected, and the spatial differentiation between microbial anomaly zones and background zones is primarily controlled by hydrocarbon abundance.

With respect to fluvial sandstone lithologic reservoirs, the microbial anomalies above the reservoirs exhibit irregular belt-like distributions. The microbial response model for lithologic reservoirs is illustrated in Figure 6B. Compared with structural reservoirs, anomalous continuity is weaker. Light hydrocarbon microseepage above lenticular lithologic reservoirs is consistent with predominantly near-vertical migration, and the distribution of hydrocarbon-oxidizing bacteria conforms to hydrocarbon accumulation patterns, with anomaly intensity potentially controlled by hydrocarbon abundance. However, because subsurface lithologic reservoirs commonly consist of multiple stacked sand bodies, surface microbial anomalies may represent the superposition of multiple reservoirs rather than a single source. Consequently, the surface anomaly geometry may not always correspond well with the shape of individual sand bodies identified by seismic data, and integrated interpretation using seismic and geological information is therefore needed.

On the basis of the microbial detection results from the eastern slope of the Fukang Depression, the microbial response model for the stratigraphic unconformity reservoirs exhibits the following characteristics. Within local areas with similar geological conditions, the distribution of hydrocarbon-oxidizing bacterial anomalies is controlled mainly by the trap geometry and hydrocarbon abundance. High-value anomalies occur as clustered or patchy distributions along unconformity surfaces, with the highest values concentrated near pinch-out zones. Anomaly intensity decreases towards structurally lower positions (Figure 6C). These results indicate that microseepage from unconformity-related reservoirs generally follows the classical light hydrocarbon microseepage model, consistent with predominantly near-vertical migration rather than lateral migration along unconformities. However, exceptions exist in areas where unconformities or faults directly connect to the surface, resulting in macroseepage. Such cases require special attention and careful discrimination from microseepage-related anomalies.

Overall, a comparison of the microbial distribution patterns above structural, lithologic, and stratigraphic reservoirs indicates that surface microbial anomalies consistent with the light hydrocarbon microseepage theory of predominantly near-vertical migration. Surface microbial responses are associated with subsurface hydrocarbon abundance and trap geometry. Under similar geological conditions, reservoir geometry and hydrocarbon abundance are the primary factors controlling both the magnitude of MV and the spatial pattern of anomalies. Consequently, analysis of surface microbial anomalies can provide a reasonable geological interpretation of subsurface hydrocarbon systems and serve as an effective tool for hydrocarbon target prediction and optimization.

### 4.2 Drilling validation of microbial responses for typical trap types

Within the Gaoquandong Anticline in Study Area A, three post-survey wells (G101, G102, and G103) were drilled in the structural low positions, all of which were located within microbial low-value zones or background zones. These wells failed to achieve the expected exploration results. Wells G101 and G103 did not encounter hydrocarbons, whereas Well G102 tested a daily oil production of 1.21 t from the Qingshuihe Formation, with a cumulative production of 11.55 t. The formation pressure coefficient of Well G102 is 2.31, and the crude oil density is 0.8471 g/cm^3^. Although Wells G102 and GT1 are both located within an abnormally high-pressure zone and exhibit similar geological conditions, only limited oil was produced from Well G102. This finding indicates that hydrocarbon abundance varies spatially and is likely controlled by structural highs. The drilling results from Well GT1 and its post-survey wells confirm a reservoir area of approximately 8 km^2^, which closely matches the extent of the microbial anomaly zone. This agreement validates the reliability of the microbial hydrocarbon detection results for predicting the spatial distribution of structural reservoirs.

For the lithologic trap type, subsequent drilling in the eastern slope of the Fukang Depression (Study Area B) included Wells FD022, FD9, FD10, and FD16. Wells FD9 and FD10 are located within microbial low-value zones or background zones, with only sporadic anomalies. The average MV (nine-point averages) above these wells are 22 CFU and 19 CFU, respectively, both below the threshold of 30 CFU, and the drilling results reveal only minor oil. In contrast, Wells FD022 and FD16 are located within microbial anomaly zones, with average microbial counts of 30 CFU and 67 CFU, respectively, exceeding the threshold, and both wells encountered commercial oil and gas.

Statistical analysis was conducted to examine the relationships between crude oil density, formation pressure coefficient, and MV (nine-point averages) for individual wells (Table 1). The crude oil densities range from 0.7488 to 0.9331 g/cm^3^, corresponding mainly to normal light oils, with only Wells FD8, FD16 and FD10 classified as medium oils, based on UNITAR classification standard (Miller, 1994). Existing drilling results indicate that Jurassic formation pressure coefficients range from 1.117 to 1.639 and that abnormal overpressure occurs in multiple producing intervals. Previous studies suggest that abnormal overpressure in the Middle Jurassic Toutunhe Formation of the eastern Fukang Depression is primarily related to Yanshanian tectonic activity, with additional contributions from sedimentary and diagenetic processes (Yu et al., 2015). Hydrocarbons preferentially accumulate near the tops of pressure-sealed compartments. Abnormal overpressure provides a driving force for hydrocarbon migration (Ma et al., 2000) and may also facilitate light hydrocarbon microseepage within reservoirs. The Cretaceous Tugulu Group acts as a regional caprock characterized by interbedded mudstones and sandstones. This lithological assemblage likely results in relatively homogeneous microseepage pathways, allowing light hydrocarbons to migrate towards the surface with a near-uniform seepage flux and to form relatively homogeneous distributions of hydrocarbon-oxidizing bacterial anomalies at the surface.

**Table 1:** Statistical summary of the drilling results and microbial values on the eastern slope of the Fukang Depression, Junggar Basin.

Well name		Pressure coefficient	Crude oil density (g/cm^3^)	Formation test result	MV (CFU)
Display wells	Well FD021	1.4604	0.8527	Cumulative production of 0.085 t from the Xishanyao Formation	8
	Well FD9	1.3605	0.8602	Daily production of 0.8 t and cumulative production of 21.16 t from the Toutunhe Formation	22
	Well FD10	–	0.9331	Daily production of 0.009 t and cumulative production of 4.25 t from the Toutunhe Formation	19
	Well FD051	1.35	0.8531	Cumulative oil production of 0.281 t from the Toutunhe Formation	22
Discovery wells	Well FD2	1.38	0.8182	Daily production of 6.67 t and cumulative production of 118.15 t from the Toutunhe Formation	32
	Well FD8	1.639	0.9115	Daily production of 5.54 t and cumulative production of 136.98 t from the Toutunhe Formation	36
	Well FD5	1.362	0.8365	Combined daily production of 38.81 t from two layers of the Toutunhe Formation, cumulative production of 865.03 t	48
	Well FD022	1.117	0.7488	Daily production of 19.4 t and cumulative production of 100.11 t from the Qigu Formation	30
	Well FD16	1.289	0.9323	Daily production of 21 t and cumulative production of 746.18 t from the Qigu Formation; daily production of 5 t and cumulative production of 139.53 t from the Sangonghe Formation	67

Regarding the validation of stratigraphic trap, a statistical analysis of surrounding wells indicated that the basal Cretaceous conglomerate generally has poor reservoir properties and is mostly dry, thereby providing partial sealing capacity. On the basis of these findings, Well FD16 was drilled to target Qigu Formation. Production testing yielded a self-flowing oil rate of 21 t/d, with a formation pressure coefficient of 1.289 and an oil density of 0.9323 g/cm^3^. Compared with Well FD8, which is located in structural low, Well FD16 has lower formation pressure and higher crude oil density, but higher production. The mean value of MV above Well FD16 is 67 CFU, while that of Well FD8 is 36 CFU, indicating that the former has higher microseepage intensity; the MV corresponds well with the hydrocarbon reservoir abundance.

According to drilling validation conducted on the seven post-survey wells, the microbial data characteristics demonstrated a 100% match with the actual drilling results. This supports that the established microbial response model can effectively predict hydrocarbon enrichment patterns in this area and enable reasonable geological interpretations of subsurface hydrocarbon reservoirs. This finding provides theoretical insights for regions with similar geological conditions. However, constrained by the validated sample size and spatial coverage, statistical uncertainty regarding this result remains, and the model’s generalizability still requires further validation through additional independent cases.

To clarify, microbial hydrocarbon exploration relies highly on the growth of near-surface microorganisms, which presents obvious limitations in some scenarios. In cases where multiple lithological hydrocarbon reservoirs overlap, the matching of microbial responses is commonly insufficient. In areas where faults distribute unevenly or where macroseepage of hydrocarbons occurs along faults, the heterogeneity of hydrocarbon microseepage fluxes will disrupt hydrocarbon-oxidizing bacteria distribution, introducing challenges in the interpretation of microbial prediction results. Meanwhile, geological factors such as reservoir pressure, hydrocarbon properties, and caprock conditions also influence the microseepage flux of light hydrocarbons, leading to “false negatives” or “false positives” in prediction results. Additionally, factors such as soil physicochemical properties, surface ecology and human disturbances, hydrogeological conditions, and seasonal climate may also influence the distribution of hydrocarbon-oxidizing bacteria (Yuan and Zhang, 2022), though these factors were not comprehensively analyzed in this study. To reduce uncertainty, their impacts could be quantitatively assessed and corrected through multivariate analysis, spatial modeling, machine learning, and molecular biology in the future (Xu et al., 2024; Zhang et al., 2024). Finally, to achieve accurate predictions of complex exploration targets, the integrated application of geomicrobial techniques with geological, geophysical, and geochemical methods is recommended. For practical application, microbial anomaly zones should be treated as priority indicator areas for screening favorable exploration targets, with monitoring signals of hydrocarbon gas concentration and composition serving as surface geochemical evidence, and geological and seismic data used to establish accumulation conditions and tectonic frameworks (Solovyeva et al., 2025; Sy et al., 2026). Together, these form a comprehensive interpretive framework that integrates hydrocarbon-oxidizing bacteria anomaly indication, hydrocarbon geochemical validation, and geological and geophysical constraints, thereby reducing the multiple-solution inherent in single-method approaches.

### 4.3 Influence of geological factors on surface microbial distributions

Previous studies have suggested that geological factors, such as reservoir pressure, hydrocarbon properties, migration pathways, and cap rock conditions, can influence light hydrocarbon microseepage fluxes, thereby affecting MV and their spatial distributions and potentially leading to false-negative or false-positive predictions. On the basis of the established relationships between microseepage characteristics and hydrocarbon-oxidizing bacterial distributions above typical reservoirs, this study investigates the effects of individual geological factors—including hydrocarbon abundance, reservoir pressure, hydrocarbon properties, migration pathways, and cap rock conditions—on the abundance of butane-oxidizing bacteria.

#### 4.3.1 Hydrocarbon abundance

Hydrocarbon abundance refers to the amount of hydrocarbons per unit area of sedimentary rock. Given that geological resources represent confidential data that is difficult to obtain, and considering the limited number of wells drilled, predictions of geological reserves are typically associated with significant uncertainty. Therefore, this study attempts to use well testing data to indicate the level of hydrocarbon abundance. Although well production rates are influenced by multiple factors—including permeability, reservoir thickness, hydrocarbon saturation, pressure, completion conditions, and test duration—Gao et al. (2021) have demonstrated that cumulative test production can reflect hydrocarbon abundance to a certain extent by establishing a mathematical relationship between test data and reserve abundance to evaluate the degree of hydrocarbon enrichment.

In the typical structural reservoir of the Sikeshu Depression, the mean MV above structurally high, hydrocarbon-rich zones (e.g., Well GT1, 179 CFU) are significantly greater than those above structurally lower zones with lower hydrocarbon abundance (e.g., Well G101: 146 CFU; G102: 148 CFU; G103: 86 CFU). Similar patterns are observed in lithologic and stratigraphic reservoirs on the eastern slope of the Fukang Depression. The mean MV above the five discovery wells are all higher than the threshold value (30 CFU), whereas the MV above the four non-commercial or display wells are consistently below the threshold. Regression analysis between cumulative test production and mean value of MV yields a significant positive correlation (n = 9, slope = 0.043, standard error = 0.008, *t* = 5.350, *p* = 0.001, Pearson’s *r* = 0.8964, *R*^2^ = 0.8035) (Figure 7A). The validation results of residual bootstrap indicate that the 95% confidence interval for *R*^2^ using BCa method is 0.6474–0.9140. Leave-one-out cross-validation shows that the predictive *R*^2^ = 0.5614, with RMSE = 10.914 and MAE = 8.544, while fitting *R*^2^ for the train set after removing a single well ranges from 0.6489 to 0.8737. Furthermore, no significant spatial autocorrelation was detected (Moran’s *I* = −0.0365, *p* = 0.740). Due to the limited sample size, these regression results carry inherent uncertainty, and the effect magnitude requires further validation with a larger sample size. Nonetheless, this association remains instructive within the available data and provides preliminary evidence of the positive relationship between both variables.

**Figure 7:**
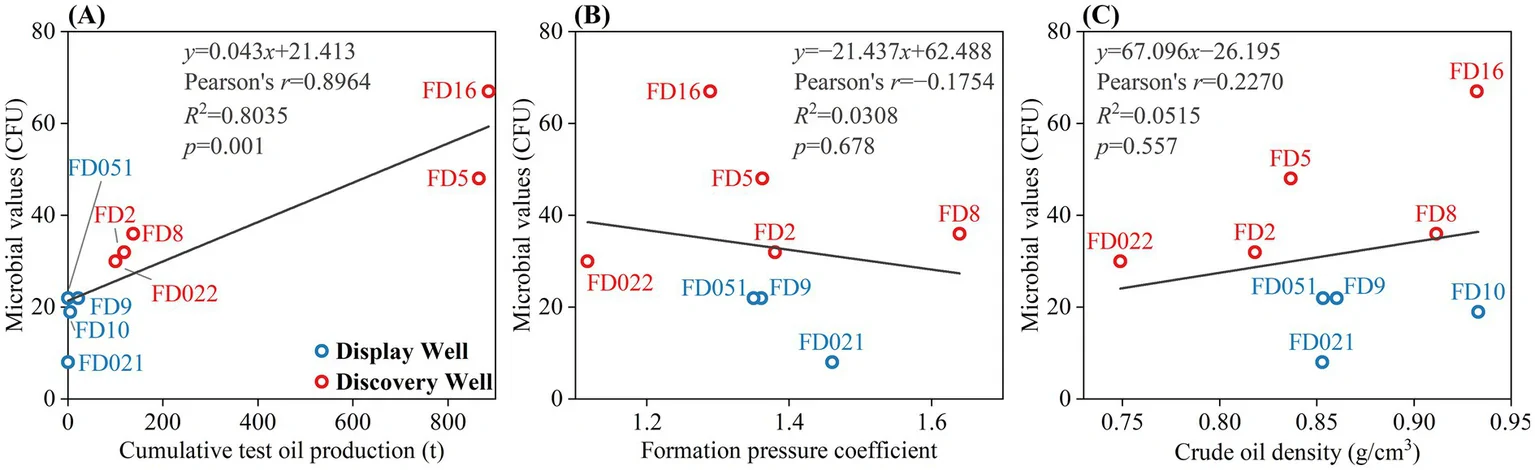
Relationships between the mean MV (Average of nine sampling points above the well) and **(A)** cumulative drilling production, **(B)** formation pressure coefficient, **(C)** crude oil density in the eastern slope of the Fukang Depression, Junggar Basin.

Hunt (1979) cited a former Soviet study showing that within several months after gas injection, gas concentrations in an aquifer located 300 m above a gas storage reservoir increased by an order of magnitude, accompanied by a significant increase in hydrocarbon-oxidizing bacteria. Zhang et al. (2010) reported similar results from comparative studies of hydrocarbon-rich and background areas in the Sichuan Basin and the Junggar Basin, where methane-oxidizing or hydrocarbon-oxidizing bacteria were consistently more abundant above hydrocarbon-rich zones.

These observations collectively indicate that high hydrocarbon abundance promotes the enrichment of hydrocarbon-oxidizing bacteria by providing sufficient carbon sources through microseepage. When hydrocarbon abundance decreases, the available of carbon sources become limited, leading to a rapid decline or disappearance of hydrocarbon-oxidizing bacteria. Thus, the strong positive correlation between cumulative test production and the mean MV may indicate that hydrocarbon abundance of subsurface reservoirs plays a significant role in driving the formation of surface hydrocarbon-oxidizing bacterial distribution patterns.

#### 4.3.2 Reservoir pressure

The upward migration of light hydrocarbons to the surface is driven not by diffusion or seepage alone but primarily by buoyancy and reservoir pressure. Liu and Sun (2007) proposed that residual pressure differences play a dominant role in both near-vertical and lateral hydrocarbon migration.

To examine the relationship between reservoir pressure and microseepage flux, reservoir pressure is represented by the pressure coefficient (the ratio of the original formation pressure to the hydrostatic pressure at the same depth), while the MV represent the microseepage intensity. Statistical analysis of these parameters was conducted for the eastern slope of the Fukang Depression (Table 1; Figure 7B). Although the MV above the discovery wells are consistently higher than the threshold, no significant correlation is observed between the MV and pressure coefficients (*n* = 8, *t* = −0.436, *p* = 0.678, Pearson’s *r* = −0.1754). The coefficient of determination is extremely low (*R*^2^ = 0.0308), much lower than the correlation between the MV and hydrocarbon abundance.

These results indicate that the original reservoir pressure is not the primary factor controlling surface MV during the exploration stage. However, previous studies have shown that MV can change dynamically during reservoir development. For example, Tucker and Hitzman (1994) reported that extensive microbial anomalies diminished after production commenced, whereas Heroy (1980) reported enhanced surface anomalies following pressure recovery during secondary development. Similar observations have been reported in Chinese basins (Yuan et al., 2001).

These findings suggest that reservoir pressure influences microseepage indirectly by affecting hydrocarbon abundance. During production, pressure depletion reduces hydrocarbon abundance and redirects pressure gradients towards wellbores, suppressing near-vertical microseepage. Conversely, pressure recovery often enhances hydrocarbon productivity and may increase microseepage intensity. Therefore, hydrocarbon abundance remains the dominant controlling factor, whereas reservoir pressure acts as an indirect influencing factor. When microbial prospecting methods are applied, wells under active development should be avoided when microbial thresholds are defined.

#### 4.3.3 Hydrocarbon properties

In the eastern slope of the Fukang Depression, crude oil densities range from 0.7488 to 0.9331 g/cm^3^, dominated by light oils, with only Wells FD8, FD10 and FD16 producing medium oil. Regression analysis between the oil density and MV reveals weak to weak positive correlations (*n* = 9, *t* = 0.617, *p* = 0.557, Pearson’s *r* = 0.2270, *R*^2^ = 0.0515; Figure 7C), indicating that the oil density is not a primary factor controlling microbial distributions under typical conditions.

Notably, among the wells producing medium oil, Wells FD8 and FD16 both achieved commercial production, with FD16 exhibiting the highest MV value among all wells, while the MV above display well FD10 was also not significantly lower than that of other display wells. This suggests that the anomalous signals from hydrocarbon-oxidizing microorganisms are not sensitive to changes in crude oil density within Study Area B, and that the differences between light and medium oils may not be sufficient to cause a significant change in the microseepage flux of light hydrocarbons. However, our hypothesis suggests that this relationship may not hold true under heavy crude oil conditions, as the extremely low content of light hydrocarbons in heavy oil could significantly weaken the enrichment of hydrocarbon-oxidizing microorganisms. But this hypothesis has yet to be empirically tested for heavy oil reservoirs, which warrants further investigation through microbial gene quantification techniques in the future (Cao et al., 2026).

#### 4.3.4 Migration pathways

The light hydrocarbon microseepage theory with predominantly near-vertical migration has long been questioned by some researchers; however, extensive field observations and experimental studies results are consistent with predominantly near-vertical migration. Wang et al. (2018) conducted a series of physical simulation experiments on light hydrocarbon microseepage and reported that microfractures serve as the primary migration pathways for hydrocarbons. Rice (1980) reported distinct light hydrocarbon anomalies directly above two, producing traps at burial depths of approximately 1,700 m, with no lateral extension beyond the oilfield boundaries. Similarly, Saunders et al. (1999) investigated an oil reservoir in Leon County, Texas, at a depth of approximately 2,680 m and reported that the lateral dispersion of soil hydrocarbon and magnetic susceptibility anomalies was less than 305 m.

A representative case was reported by GMT (Geo-Microbial Technologies) during the exploration of Cotton Valley pinnacle reefs in Texas (Hitzman et al., 1999). The Jurassic marine pinnacle reef gas reservoirs are extremely small (0.08–0.32 km^2^), deeply buried (4,300–5,400 m), overpressured, and sealed by nearly 1,000 m of anhydrite caprock. Microbial survey results revealed that high-production gas flows were obtained from two reefs associated with strong microbial anomalies, with daily gas production ranging from 4.2 × 10^5^ to 8.49 × 10^5^ m^3^, whereas no commercial gas flow was encountered in the two reefs located within the areas with a background microbial activity. The successful prediction of such small lithologic traps further confirms that light hydrocarbon migration during microseepage aligns with predominantly near-vertical rather than diffusive.

Light hydrocarbon microseepage occurs mainly through micron- to nanoscale microfractures that are widely distributed within strata. These microfractures provide effective migration pathways for hydrocarbons to escape upwards under the combined effects of buoyancy and formation pressure, resulting in the development of “apical anomalies” above subsurface reservoirs. Nevertheless, hydrocarbons may also migrate laterally along unconformities or faults, producing macroscopic seepage (“macroseepage”), which is fundamentally different from microseepage in both mechanism and expression.

Microseepage characteristics of hydrocarbons migrating upward through pervasive microfractures into surface soils or seabed sediments are consistent with predominantly near-vertical migration. The hydrocarbon concentrations associated with microseepage are typically less than 200 ppm and are dominated by low-carbon-number alkanes (C₆^−^), with little to no C₆^+^ heavy hydrocarbons. The resulting anomalies of hydrocarbon-oxidizing bacteria at the surface generally correspond well to the underlying reservoirs and commonly appear as clustered, patchy, or banded patterns.

In contrast, macroseepage refers to the lateral migration of hydrocarbons along preferential pathways such as regional unconformities or active fault zones. Rosaire (1940) first recognized the distinction between macroseepage and microseepage, which was further discussed by Jones and Drozd (1983) and Price (1985). Abrams (2005) provided a systematic definition of macroseepage, describing it as the visible flow of high-concentration hydrocarbons (Darcy flow). In macroseepage, both light hydrocarbons (C₆^−^) and heavy hydrocarbon components can migrate to the surface. Hydrocarbon concentrations associated with macroseepage are several times higher than those associated with microseepage and may exceed 10,000 ppm, typically forming linear anomalies along faults or unconformities and manifesting as mud volcanoes or oil and gas seeps.

In summary, light hydrocarbon microseepage controlled by microfracture networks generally exhibits hydrocarbon fluxes lower than 200 ppm and produces apical microbial anomalies that are governed primarily by reservoir abundance and trap geometry. In contrast, hydrocarbon macroseepage along faults or unconformities is characterized by much higher fluxes, which typically exceed 1,000 ppm, and generates linear microbial anomalies that are indicative of macroscopic seepage. These two seepage modes should be clearly distinguished in hydrocarbon prediction and exploration studies.

#### 4.3.5 Caprock conditions

Caprocks are commonly composed of mudstone, shale, evaporites (gypsum and halite), and dense limestone. Although caprocks play a critical role in sealing hydrocarbons and controlling reservoir accumulation, their presence does not necessarily prevent the upward migration of light hydrocarbons. Experimental studies by Davidson (1963) and Duchscherer (1981) demonstrated that inert gases and hydrogen can diffuse through the atomic structures of metals and glass, leading to the conclusion that “nothing is absolutely impermeable.”

In the microbial hydrocarbon prediction case from the Gaoquandong Anticline in Study Area A, the Cretaceous Tugulu Group serves as a regional caprock composed mainly of mudstone with a thickness of 500–2,000 m. Despite the presence of this thick regional caprock, pronounced microbial anomalies were still detected at the surface. This phenomenon may be attributed to the migration of light hydrocarbons through microfractures in caprocks (Hunt, 1979). In addition, light hydrocarbons are also affected by geological conditions such as unconformities and faults to cause macroseepage (Abrams, 2005). Although no instance of macroseepage was observed in this study, this phenomenon must be distinguished in the field application of microbial hydrocarbon exploration. However, previous studies have also shown that the heterogeneous distribution of thick evaporite caprocks may significantly influence microbial survey results and lead to apparent false-negative anomalies. Beghtel et al. (1987) reported large-scale microbial hydrocarbon surveys conducted in Kansas, USA, where microbial prediction results in evaporite-bearing areas differed substantially from drilling outcomes. The primary reason was the presence of thick evaporite caprocks, which reduced the intensity of light hydrocarbon microseepage in the western region compared with that in the eastern region, where evaporites were absent. When hydrocarbon-oxidizing bacterial values from both regions were statistically analysed together, false-negative results were produced in evaporite-dominated areas.

In summary, light hydrocarbons can migrate to the surface even when present with dense caprock lithologies, including mudstone, shale, evaporites (gypsum and halite), and even dense limestone. In regions where regional caprocks are relatively uniform in distribution, the influence of caprocks on surface hydrocarbon anomalies is limited, and anomaly patterns are controlled mainly by hydrocarbon abundance and trap geometry. In contrast, in areas where dense caprocks such as evaporites are well developed but heterogeneously distributed, significant variations in microseepage intensity may occur, which should be carefully considered when interpreting microbial survey results.

## 5 Conclusion

The field investigation in the Junggar Basin reveals the relationships between geological conditions and microbial responses above typical hydrocarbon reservoirs. Hydrocarbon-oxidizing bacterial anomalies above anticlinal reservoirs are predominantly concentrated in the structural core and exhibit clustered patterns. In contrast, the anomalies above the stratigraphic unconformity reservoirs are distributed mainly along unconformity surfaces in clustered or sheet-like forms, whereas those above the lithologic reservoirs exhibit irregular belt-shaped distributions. The spatial distribution patterns of microbial anomalies above different reservoir types are consistent with the theory that light hydrocarbon microseepage predominantly migrates near-vertically. The anomalous distribution of surface hydrocarbon-oxidizing bacteria is strongly correlated with the subsurface accumulation of hydrocarbons. During exploration, analysing the distribution characteristics of hydrocarbon-oxidizing bacterial anomalies in combination with microbial response–geological models enable reasonable geological interpretation of microbial anomalies and supports effective prediction and prioritization of hydrocarbon-bearing targets. But this method cannot independently determine the depth or stratigraphic position of hydrocarbon reservoirs. Therefore, microbial exploration should be integrated with geological, geophysical, and geochemical methods to compensate for its limitations in depth prediction. A multidisciplinary integrated exploration framework is expected to provide an effective new approach for evaluating hydrocarbon potential in complex exploration settings.

## Data Availability

The original contributions presented in the study are included in the article/Supplementary material, further inquiries can be directed to the corresponding author.
