# Supplementary material for: Controls on microbial response above typical oil and gas reservoirs in the Junggar Basin and implications for exploration
Source: Front Microbiol. 2026 Oct 21;17:1979085. doi: 10.3389/fmicb.2026.1979085 (PMC13732493; doi:10.3389/fmicb.2026.1979085)
Supplement: Supplementary file 1 [file Table_1.DOCX]

Supplementary Material

# 1 Supplementary Table

**Supplementary Table 1. Summary of relevant data pertaining to the wells within the study area of Junggar Basin**

| **Well name** | **Drill completion time** | **Types of well** | **Sampling time** | **Production status during sampling** | **Distance between sampling points and the well (m)** | **Average microbial value (CFU)** | **Consistency between microbial detection and drilling results** |
| --- | --- | --- | --- | --- | --- | --- | --- |
| GT1^a^ | 2018.11 | Known well, Discovery well | 2018.07 | Drilling in progress | 230–960 | 179 | Consistent |
| G101 | 2020.04 | Post-survey well, Display well | 2018.07 | Undrilled | 270–990 | 146 | Consistent |
| G102 | 2020.02 | Post-survey well, Display well | 2018.07 | Undrilled | 230–930 | 148 | Consistent |
| G103 | 2020.11 | Post-survey well, Display well | 2018.07 | Undrilled | 310–1020 | 86 | Consistent |
| FD2 | 2010.09 | Known well, Discovery well | 2011.08 | In production | 70–505 | 32 | Consistent |
| FD021 | 2011.03 | Known well, Display well | 2011.08 | Shut-in | 135–650 | 8 | Consistent |
| FD5 | 2011.03 | Known well, Discovery well | 2011.08 | In production | 175–610 | 48 | Consistent |
| FD051 | 2011.08 | Known well, Display well | 2011.08 | Shut-in | 190–660 | 22 | Consistent |
| FD8 | 2011.07 | Known well, Discovery well | 2011.08 | In production | 130–525 | 36 | Consistent |
| FD9 | 2011.09 | Post-survey well, Display well | 2011.08 | Drilling in progress | 130–700 | 22 | Consistent |
| FD10 | 2011.10 | Post-survey well, Display well | 2011.08 | Drilling in progress | 150–700 | 19 | Consistent |
| FD022 | 2011.09 | Post-survey well, Discovery well | 2011.08 | Drilling in progress | 130–575 | 30 | Consistent |
| FD16 | 2012.05 | Post-survey well, Discovery well | 2011.08 | Undrilled | 180–680 | 67 | Consistent |

^a^ Hydrocarbon discovery in Well GT1 was already known upon sampling.
